# Posttraumatic Ulnar Translocation of the Carpus: A Case Report and Brief Review of the Literature

**DOI:** 10.1016/j.jhsg.2021.10.002

**Published:** 2021-11-05

**Authors:** Matthew Nasra, Vivian Chen, David Kirschenbaum, Brian M. Katt

**Affiliations:** ∗ Rutgers Robert Wood Johnson Medical School, New Brunswick, NJ

**Keywords:** Ligament avulsion, Posttraumatic, Ulnar translocation, Volar fleck, Wrist injury

## Abstract

We report a case of posttraumatic ulnar translocation of the carpus, which resulted after a fall from a six-foot ladder. This patient presented with multiple injuries to the skull bones, face, and limbs. A diagnosis of ulnar translocation of the carpus was missed on initial radiographs. Ulnar translocations require a high clinical index of suspicion and should be considered in the context of any high-impact injury to the wrist. A volar fleck just distal to the radial articular surface represents evidence of ligamentous disruption and should alert physicians that a more severe injury may be present. Nonsurgical and surgical treatment options are reviewed.

Ulnar translocation (UT) of the carpus is a rare pattern of radiocarpal instability. It has been described with degenerative wrist changes in patients with rheumatoid arthritis.^1^^,^^2^ Ulnar translocations of the carpus have also been reported following trauma. Posttraumatic ulnar translocation (PTUT) is an infrequent event that is often missed on initial presentation.^2^, ^3^, ^4^, ^5^, ^6^ As a result, there can be a considerable delay in diagnosis and treatment with poorer outcomes.^3^ An optimal treatment method has not yet been established. Here, we report a case of PTUT in a 71-year-old-man diagnosed 7 weeks after the initial injury. Conservative treatment was successful in managing symptoms.

## Case report

A 71-year-old right-handed man presented with multiple injuries following a mechanical fall from a ladder sustained while hanging Christmas lights. The patient fell forward onto his face and outstretched hands. He sustained various facial fractures that were emergently treated. Additionally, he complained of left wrist pain.

Initial examination revealed the left upper extremity to be neurovascularly intact, swelling about the base of the first metacarpal joint, and tenderness to palpation over the anatomical snuffbox. Initial plain radiographs of the left wrist were read as normal. The patient’s left upper extremity was placed in a thumb spica splint for a suspected nondisplaced scaphoid fracture, and he was told to follow up outpatient.

**Figure 1:**
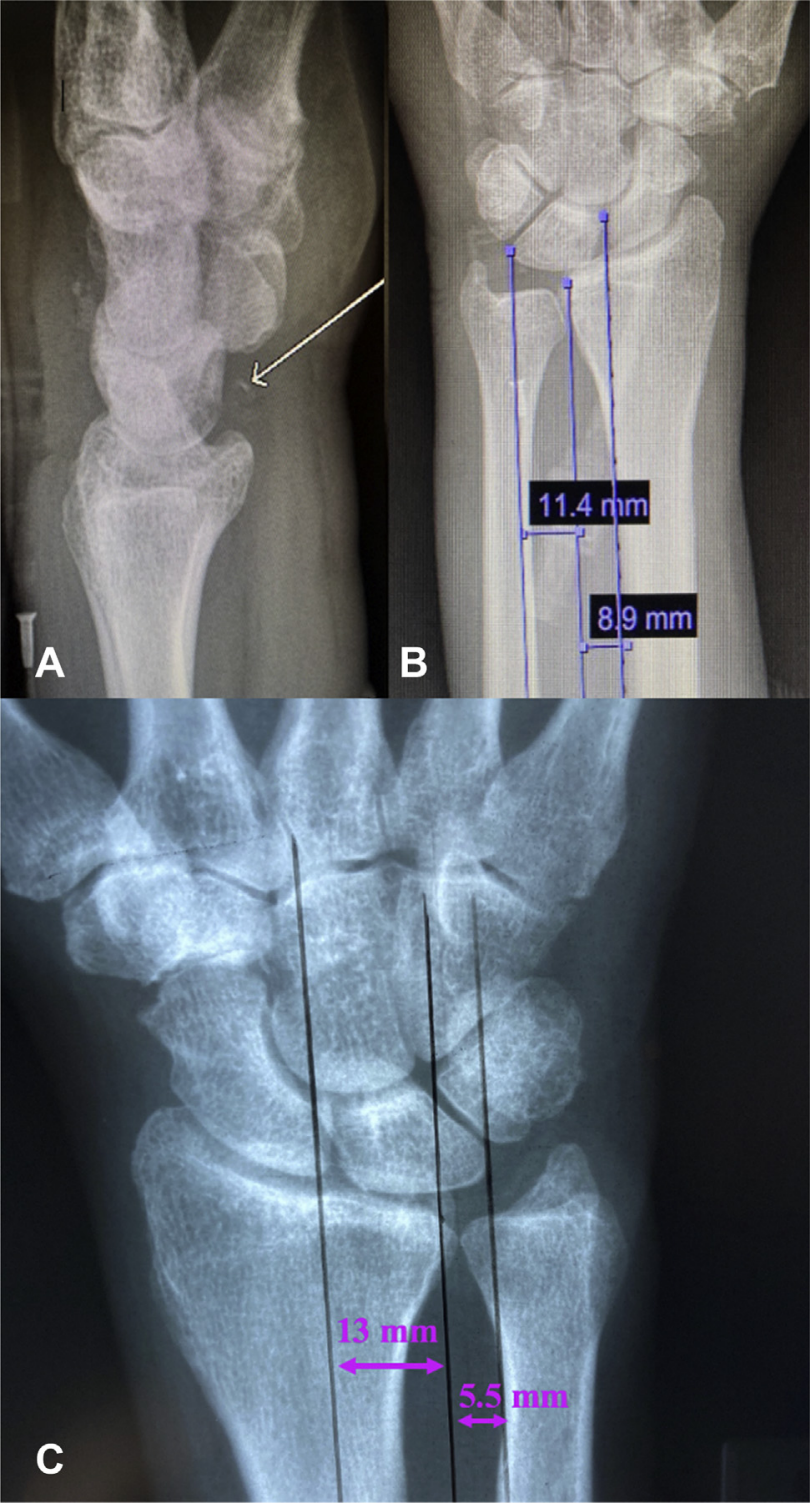
Plain radiographs of the injured and uninjured wrist**. A** Lateral view of the left wrist. Volar fleck is identified by the white arrow**. B** PA view of the left wrist. Lunate uncovering 56%. **C** PA view of the contralateral wrist. Lunate uncovering 30%.

**Figure 2:**
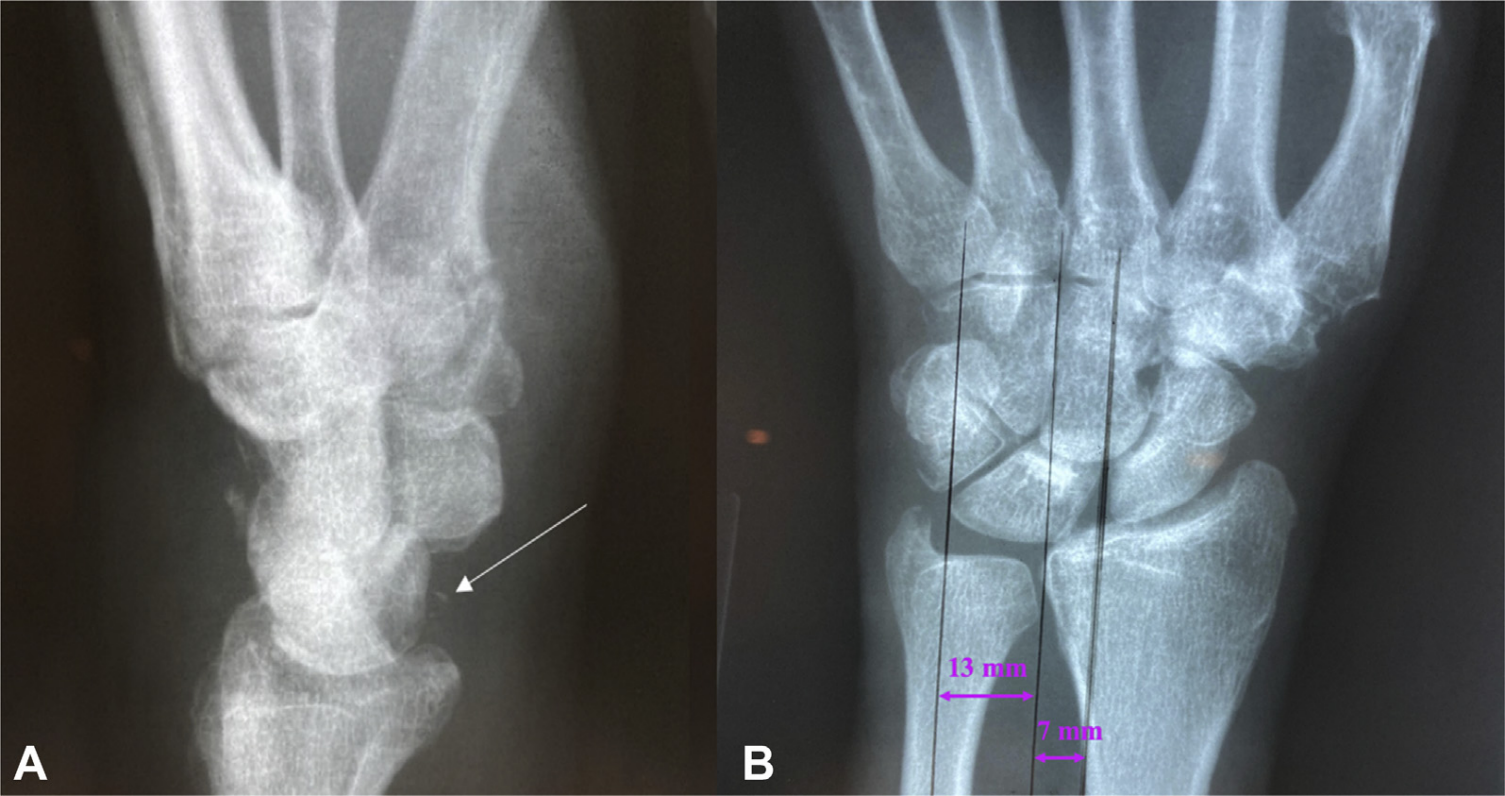
Radiographs of the left wrist taken at 7 weeks after the initial injury. **A** Lateral view. Volar fleck is identified by the white arrow. **B** PA view. Lunate uncovering 65%.

At the 7-week follow-up visit, the patient had continued mild left-sided wrist pain, which prompted a reevaluation of the initial radiographs. A retrospective diagnosis of volar ligament injury was made. A small volar fleck fracture was associated with the ulnar translation of the proximal carpal row (Fig. 1). The hand attending physician, hand surgery resident, and radiologists missed this diagnosis on the initial presentation. New radiographs taken at this visit demonstrated that the entire carpus had translocated ulnarly (Fig. 2). Because of the minimal symptoms and delay in diagnosis, he continued to be managed nonsurgically.

At the 14-week follow-up, he again had minimal pain and a slight cosmetic ulnar deformity. Wrist examination revealed 60° of extension, 45° of flexion, 25° of ulnar deviation, 10° of radial deviation, symmetrical rotation to the uninjured side, and a full fist.

**Figure 3:**
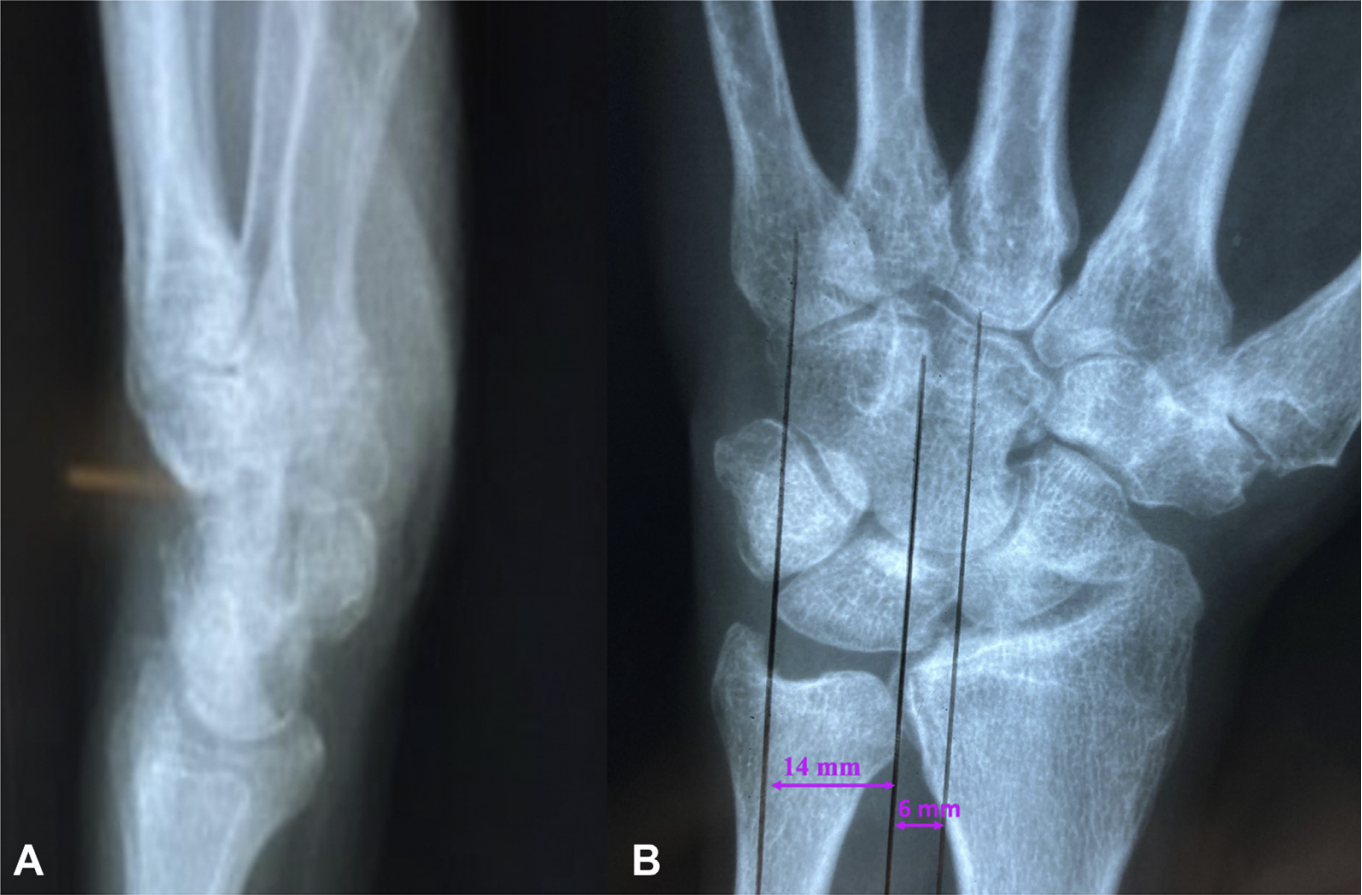
Radiographs of the left wrist taken at 6 months after the initial injury. **A** Lateral view. **B** PA view. Lunate uncovering 70%.

At 6 months, the patient denied any pain symptoms. He used a splint when he occasionally did yard work but otherwise did not use immobilization. Examination of the affected wrist revealed 60° of wrist extension, 50° of wrist flexion, 25° of ulnar deviation, 10° of radial deviation, and the contralateral wrist had 80°, 70°, 40°, 20°, respectively. Grip strength at level 3 of the Jamar dynamometer was 18.1 kgf on the injured side and 29.5 kgf on the uninjured side. There was no evidence of functional carpal instability or degenerative radiographic changes (Fig. 3). At 10 months postinjury, the patient was contacted by phone. He mentioned having mild pain with heavy activities such as yard work. However, using a cock-up wrist splint while doing strenuous activities allowed him to work without pain. He takes no pain medication for his wrist and is satisfied with his current upper extremity function.

## Discussion

Posttraumatic UT occurs because of severe and global disruption to the ligamentous structures of the wrist and is defined as any abnormal translation of the lunate in the ulnar direction.^1,7^ The mechanism of injury is likely due to hyperextension, ulnar deviation torque, or pronation on a fixed hand.^3^^,^^4^ When there is a severe disruption to the radioscaphocapitate, ulnolunate, and radiolunate ligaments that restrain the carpus, the carpus translates ulnarly along the radius.^1^ Taleisnik^7^ has described 2 types of UT. In type 1 injuries, the entire carpus translates, and the space between the scaphoid and radial styloid widens. While in type 2 injuries, the scaphoid and radial styloid distance remains, and the scapholunate space widens.^7^ The injury pattern in our patient had characteristics of both type 1 and 2 injuries.

Patients with PTUT typically present with pain, swelling, functional impairment, and ulnar wrist deformity.^3^^,^^5^ On radiographs, UT can be identified as a widening of the radiocarpal joint at the radial styloid process, when less than 50% of the lunate articulates with the radius in neutral posteroanterior (PA) radiograph, an increase in the distance between the radial styloid and scaphoid, or an abnormal positioning of the scaphoid in the lunate fossa.^2^^,^^4^

Ulnar translocation can be quantified by determining the carpal-ulnar distance ratio or the lunate uncovering percentage. A normal carpal-ulnar distance ratio measures 0.3 ± 0.03. When this ratio is <0.27, or there is a >0.03 difference between the ratios of the injured and uninjured wrists, a UT can be diagnosed.^5^^,^^8^ Alternatively, lunate uncovering uses the distance between the farthest point on the ulnar border of the lunate and a line drawn through the ulnar edge of the lunate facet divided by the full width of the lunate and expressed as a percentage. A normal lunate uncovering is 37% to 44%, and UT is defined as >44% of the lunate medial to the lunate facet.^5^^,^^8^ In our case, the Schuind method was used to measure lunate uncovering.^9^

When UT occurs in the presence of trauma, it may be masked on initial presentation. The extent of trauma required to disrupt the carpal ligaments results in concomitant injuries that take priority in treatment and may contribute to a delay in diagnosis.^3^^,^^5^^,^^10^ In our patient, his multiple facial fractures were treated first, which may have resulted in a less thorough examination of the wrist. Additionally, the deformity of PTUT tends to progress over time.^2^^,^^3^ In this case, the lunate translation progressed over one month; the initial lunate uncovering measurement was 56%, and the percentage increased to 65% by 4 weeks. At 6 months, the lunate uncovering measured 70%.

Initial radiographs may show minimal displacement. Therefore stress views with a perpendicular ulnar-directed force can help determine the diagnosis.^2^^,^^5^ The uninjured wrist should be imaged for comparison. In our patient, stress views and contralateral views were not taken at the time of injury. These may have aided in making a timely diagnosis of UT.

Clinicians require a high index of suspicion to diagnose UT properly. Evidence of ligamentous disruption can demonstrate that a more severe injury is present. The volar fleck, volar to the scaphoid, represented an avulsion fracture from the volar lip of the radius and was identified easily on the initial radiographs. Prior case reports of PTUT have been associated with avulsion fractures of the distal radius.^5^^,^^11^ When present, a volar fleck is likely pathognomonic for ligament avulsions. Patients with this finding should be investigated for UT and carpal instability. Magnetic resonance imaging can be valuable to characterize the extent of soft-tissue damage. However, advanced imaging is dependent on surgeon preference.^5^ In our case, magnetic resonance imaging was ordered, however, the quality was poor as the patient could not tolerate the procedure.

The delay in diagnosis can result in suboptimal outcomes. Rayhack et al^3^ reported 8 cases of PTUT with an average time to diagnosis of 7.3 months after initial injury treated with ligamentous repair and radiocarpal pinning. Seven patients experienced recurrent UT, with 3 requiring secondary wrist arthrodesis and 1 resulting in degenerative arthritis.^3^ Rutgers et al^4^ described 2 cases of PTUT diagnosed 2 months after the initial injury treated with ligamentous repair and K-wire stabilization. One patient experienced discomfort with repetitive wrist motion and recurrent ulnar drift but no interval arthrosis, while the other never fully recovered wrist extension/flexion and grip strength.^4^

Earlier recognition may provide adequate results. Options for surgical treatment include dorsal and/or volar radiocarpal ligament repair, tendon augmentation of ligament repairs, radiocarpal pinning, fracture fixation, radiolunate arthrodesis, and complete wrist arthrodesis.^2^^,^^3^^,^^5^^,^^7^^,^^10^^,^^11^ Berschback et al^5^ described 10 cases of PTUT treated with ligamentous repair and/or wrist fracture stabilization within 2 months of injury. Recurrent ulnar translation occurred in 9 of 10 patients. Six developed wrist arthroses, yet only 1 required secondary radiolunate fusion. All 6 patients who returned for follow-up resumed preinjury work activities, with an average Disabilities of the Arm, Shoulder, and Hand score of 6, and Mayo modified wrist score of 76.^5^ Innocenti et al^6^ also documented a case of PTUT treated with volar ligament repair 1 month after the initial injury. The patient remained pain-free, had a Disabilities of the Arm, Shoulder, and Hand score of 4.2, and did not experience recurrence of UT or degenerative changes.

While earlier recognition is crucial, appropriate treatment choice is equally critical for successful outcomes. Bellinghausen et al^11^ reported 2 cases of PTUT and palmar carpal subluxation treated nonsurgically with cast immobilization after closed reduction. This treatment was unsuccessful as both patients experienced recurrent UT, palmar radiocarpal subluxation, degenerative changes, and remodeling deformities.^11^ Stäbler et al^2^ presented 2 cases of PTUT with rotatory palmar subluxation of the lunate. One patient was treated conservatively, which resulted in recurrent UT, severely limited range of motion, and abnormal articulation of the lunate with the radius. Another patient diagnosed 4 days after injury was treated with ligament repair and radiocarpal fixation. The patient had normal function, minimal restrictions in wrist motion, no degenerative changes, but persistent UT.^2^ Jebson et al^10^ presented a case of PTUT diagnosed 3 weeks after the initial injury was treated with K-wire stabilization and external fixation. This patient continued to have instability and ultimately required radiolunate arthrodesis. There was no degenerative change at the midcarpal joint, and the patient was pain-free and recovered his grip strength by 14-months.^10^

PTUT is challenging to treat, and there are no official treatment guidelines. Conservative management, with observation, closed reduction, and cast immobilization, have largely proven ineffective.^2^^,^^5^^,^^11^ Operative treatments have had better results, although most cases experienced recurrent ulnar translation or developed wrist arthritis.^2^, ^3^, ^4^, ^5^, ^6^^,^^10^ In our case, conservative care resulted in a satisfactory short-term outcome. Traumatic arthritis may progress over time, and the patient may require reconstruction. We elected not to fuse him at this stage given that he had good motion, was pain-free, and was low demand. Limited arthrodesis can be performed at a later date should pain that impairs function develop.

Clinicians should maintain a high index of suspicion for PTUT in the setting of high-energy injuries with signs of ligamentous injury on imaging, such as the volar fleck sign, and strongly consider contralateral imaging. Surgical repair is favored in cases diagnosed acutely. Conservative management may be considered for chronic cases if the patient presents with minimal symptoms, particularly if elderly and low demand. Otherwise, ligamentous reconstruction or limited wrist fusion may be warranted.
